# Mitochondrial Serine Protease HTRA2 p.G399S in a Female with Di George Syndrome and Parkinson's Disease

**DOI:** 10.1155/2018/5651435

**Published:** 2018-06-21

**Authors:** Stefano Gambardella, Rosangela Ferese, Simona Scala, Stefania Carboni, Francesca Biagioni, Giardina Emiliano, Stefania Zampatti, Nicola Modugno, Francesco Fabbiano, Francesco Fornai, Diego Centonze, Stefano Ruggieri

**Affiliations:** ^1^IRCCS Neuromed, Pozzilli, Italy; ^2^Molecular Genetics Laboratory UILDM, Santa Lucia Foundation, Rome, Italy; ^3^Department of Biomedicine and Prevention, University of Rome “Tor Vergata”, Rome, Italy; ^4^Department of Translational Research and New Technologies in Medicine and Surgery, University of Pisa, Pisa, Italy

## Abstract

Deletion at 22q11.2 responsible for Di George syndrome (DGs) is a risk factor for early-onset Parkinson's disease (EOPD). To date, all patients reported with 22q11.2 deletions and parkinsonian features are negative for a family history of PD, and possible mutations in PD-related genes were not properly evaluated. The goal of this paper was to identify variants in PD genes that could contribute, together with 22q11.2 del, to the onset of parkinsonian features in patients affected by Di George syndrome. To this aim, sequencing analysis of 4800 genes including 17 PD-related genes was performed in a patient affected by DGs and EOPD. The analysis identified mutation p.Gly399Ser in *OMI/HTRA2* (PARK13). To date, the mechanism that links DGs with parkinsonian features is poorly understood. The identification of a mutation in a PARK gene suggests that variants in PD-related genes, or in genes still not associated with PD, could contribute, together with deletion at 22q11.2, to the EOPD in patients affected by DGs. Further genetic analyses in a large number of patients are strongly required to understand this mechanism and to establish the pathogenetic role of p.Gly399Ser in *OMI/HTRA2*.

## 1. Introduction

The 22q11.2 deletion syndrome (22qDS), also known as Di George syndrome (DGs), is a multisystem disorder caused by a chromosomal microdeletion most commonly involving a 3 Mb segment on the long arm of chromosome 22. The hallmark features of the syndrome consist of typical facial appearance, velopharyngeal failure, conotruncal heart disease, parathyroid and immune dysfunction, developmental delays, and learning difficulties. Late-onset complications, including recurrent seizures and schizophrenia, are common [1]. In a considerable amount of patients carrying 22qDS, early-onset, nonfamilial Parkinson's disease (EOPD) is described which represents the focus of the present report.

Parkinson's disease (PD) is a neurodegenerative movement disorder characterized by tremor, muscle rigidity, bradykinesia, gait disturbances, and a variety of nonmotor symptoms. The life-time risk for developing PD is 1-2% [2]. PD typically occurs late in life, but 4% of cases has an onset before the age of 50 [3].

Early neurological reports described the occurrence of movement disorders in 22qDS, which were already evident in childhood and adolescence [4]. These extrapyramidal motor symptoms were initially interpreted as a consequence of antipsychotic medication [5]. The idea that parkinsonian symptoms in 22qD patients occur specifically as the consequence of nigrostriatal degeneration has been proposed years later when two 22qDS patients without antipsychotic medications were found to develop EOPD (age < 45 years) [6]. Nonetheless, the association between 22qDS and PD remained at the anecdotal level. Later on, a case-control study confirmed a high prevalence of PD in 22qDS patients. Very recently, a large study including more than 9,000 PD patients indicates that 22q11.2 deletions lead to 20-fold increased risk to develop EOPD [7]. This report, despite highlighting a strong epidemiological evidence, it does not provide a genetic investigation of PD-related genes apart from the 22q11.2 mutation. Moreover previous studies analyzing a few patients, report only very few or no genetic analysis related to PD [8, 9].

In an effort to correlate at the molecular level the clinical association between DGs and EOPD, in the present study, we report a detailed genetic study carried out by analyzing the clinical exome including 18 PD-related genes in a woman with a clinical diagnosis of DGs confirmed by the presence of a 22q deletion.

## 2. Materials and Methods

Genomic DNA was isolated from peripheral blood leukocytes according to standard procedures. Clinical exome sequencing considering 4800 human genes including 18 genes related to Parkinson's disease (PARK1: *SNCA*; PARK2: *PRKN*; PARK3: *SPR*; PARK4: *SNCA* Dup/Del; PARK5: *UCHL1*; PARK6: *PINK1*; PARK7: *DJ1*; PARK8: *LRRK2*; PARK9: *ATP13A2*; PARK10: *ELAVL4*; PARK11: *GIGYF2*; PARK12: *TAF1*; PARK13: *HTRA2*; PARK14: *PLA2G6*; PARK15: *FBXO7*; PARK16: *ADORA1*; PARK17: *VPS35*; PARK18: *EIF4GI*) (TruSight One Sequencing Panels, Illumina) were performed on MiSeq platform (Illumina). Variant Studio was used for annotation and characterization of variants. The manual examination and visualization of the sequence data were performed by the Integrative Genomics Viewer v.2.3, while selection of potentially pathogenetic variant by Tgex software (LifeMap Sciences). Mutations were resequenced by Sanger sequencing (ABI 3130xl Genetic Analyzer, Applied Biosystems).

## 3. Results

The woman reported in the present study had a clinical diagnosis of DGs confirmed by the presence of a 22q deletion. Specifically, dysmorphological features were recorded (hypertelorism, low-set ears, abnormal folded pinna, short philtrum, micrognathia, and hypoacousia). The mother and her 9-year-old son were also affected by DGs.

She came to our hospital in 2011, when she was 35 years old, due to the onset of extrapyramidal tremor of the left hand which was a slight progressive start up at 12 months before hospital admission. This symptom further progressed to impair fine motor control which deteriorated up to a loss of dexterity in the left hand. She had a hypomimic face and suppressed blinking. She was bradykinetic, and swinging while walking was reduced much more in the left than the right side. She suffered from muscle rigidity which was prevalent in the upper left limb.

**Figure 1:**
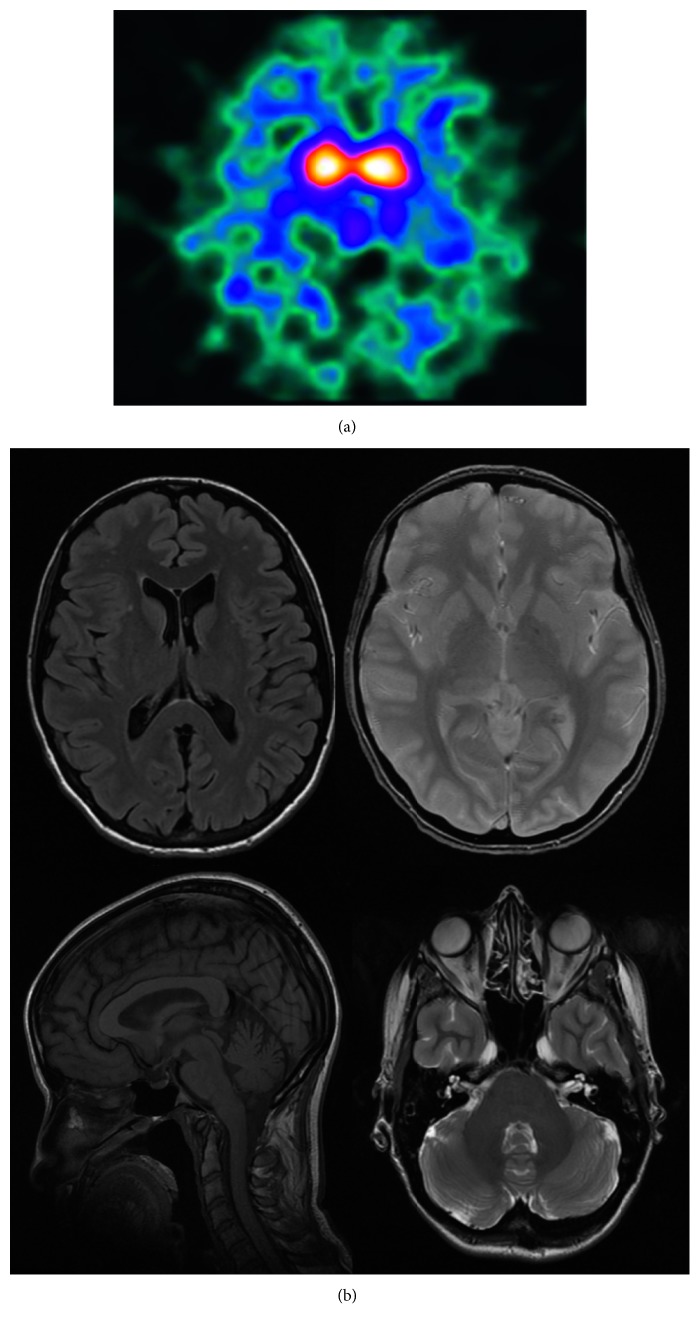
DaTSCAN and magnetic resonance imaging. (a) DaTSCAN image showing a reduced tracer uptake in both striatal regions, especially on the right side. (b) Magnetic resonance imaging (MRI) scan showed a normal profile with aspecific gliotic microvascular outbreaks in subcortical white matter of front-insular hemispheres. Axial Flair MRI scan shows aspecific gliotic microvascular outbreaks in subcortical white matter of front-insular hemispheres; Axial GRE MRI scan shows normal putamina bilaterally in absence of degeneration signs or deposits; sagittal-T1 MRI scan shows normal midbrain; axial-T2 MRI scan shows normal cerebellum.

SPECT scan using the radioisotope [123I]-beta-CIT showed a decreased dopamine (DA) uptake bilaterally in the putamen (Figure 1(a)). Magnetic resonance imaging (MRI) scan showed a normal profile with aspecific gliotic microvascular outbreaks in subcortical white matter of front-insular hemispheres (Figure 1(b)).

IQ evaluation revealed a value under the mean. In fact, the WAIS-R (Wechsler Adult Intelligence Scale-Revised; Wechsler, 1981) revealed (i) verbal IQ: 72/100, (ii) performance IQ: 70/100, (iii) total IQ: 70/100 points (Wechsler and Ogdon, 1981); the MMPI (Minnesota Multiphasic Personality Inventory) showed Pd score: 73/100 and Pa: 75/100. Laboratory exams revealed hypocalcemia (7.3 mg/dl), hyperprolactinemia (she was under oral estroprogestinic anticontraceptive therapy), mild hypercholesterolemia (209 mg/dl), and increased ESR (65 mm). The clinical and ultrasonographic cardiological evaluation revealed a mild tricuspid and mitral valves regurgitation with 66% ejection fraction. Audiometry was normal.

In 2013, [^18^F] DOPA PET scan revealed decreased uptake in the right putamen. The UPDRS-III scored 57 and 27 before and after levodopa administration, respectively, and 21 following apomorphine.

In 2015, neuropsychological evaluation confirmed previous results both at WAIS-R and at MMPI. Hypocalcemia was still present. After administration of 250 mg of levodopa and 25 mg of carbidopa, the patients showed dyskinesia and dystonia at the left lower arm.

At this time, molecular genetic evaluation was carried out. Clinical exome sequencing identified the mutation p.Gly399Ser (c.1195G>A, NM_013247.4) in *OMI/HTRA2* (OMIM #606441), recently designated as Parkinson disease-13 locus (PARK13). No mutations in other PD-related genes was identified (Table 1). This variant was detected also in the mother, who did not show either movement disorder or other neurological deficit.

## 4. Discussion

To date, less than 20 patients carrying 22q11.2 deletions and PD have been reported worldwide [6–10]. Unfortunately, in all of them, a whole genetic analysis for PD is missing despite all patients reporting EOPD symptoms (age at onset ranging between 39 and 48 years) [10]. Most of them were treated with dopamine replacement therapy (L-DOPA), with some initial improvement in motor symptoms quickly followed by motor fluctuations and dyskinesia [8–10]. Psychotic symptoms are frequent with anxiety and depressed mood up to suicide attempts. Degeneration of the nigrostriatal dopamine system has been reported in vivo by presynaptic dopamine imaging, and it is confirmed at brain autopsy showing PD neuropathology which was suggested to be quite reminiscent of LRRK2-associated PD [10, 11].

To date, all patients carrying concomitantly 22q11.2 deletions and parkinsonian features were negative for a family history of PD and any known pathogenic PD-related mutations. However, the latter were poorly investigated. This is the first report of a 22q11.2 patient affected by PD where a dedicated next generation sequencing analysis for PD genes was carried out. This extensive approach allowed detecting a specific mutation affecting a gene known to be involved in PD.

The PARK13 locus, OMI/HTRA2 (OMIM #606441), encodes a serine-protease with proapoptotic activity and a mitochondrial targeting sequence placed at its N-terminal region. Despite the existing controversial data [12–21], several findings suggest a potential link between *OMI/HTRA2* and PD [12, 13]. In fact, *Omi/HtrA2* knockout or transgenic mice possess a phenotype either reminiscent of PD or motor neuron disease, respectively. Remarkably, in vitro Omi/HtrA2 interacts with PINK1 which is known to sense and regulate mitochondrial alteration and removal [14–17].

Based on these intriguing links between *Omi/HtrA2* and PD, *Omi/HtrA2* was recently designated as *Parkinson disease-13* locus (*PARK13*). Mutations in *OMI/HTRA2* were associated with both PD and essential tremor, and these data are strengthened by the presence of Omi/Htra2 in Lewy bodies in brains of idiopathic PD patients [12, 18]. In particular, the variant p.G399S leads to mitochondrial dysfunction, altered mitochondrial morphology, and decreased protease activity.

Thus, the pathological role of p.G399S in PD still has to be fully understood concerning its pathogenicity and penetrance. We suggest that, in patients with 22qdel, this variant increases the risk to develop EOPD. In fact, variants that confer an improved or decreased risk to PD and variants with a reduced penetrance are a common feature of neurodegenerative disorders, including several PD loci (e.g., *LRRK2*, *SNCA*, and *VPS35*) [22].

This is compatible with the presence of the p.G399S variant in the proband's mother, who did not show either movement disorder or other neurological deficit. The genetic test in the son of the proband is mandatory to improve our knowledge on the penetrance of this mutation. Unfortunately, the mother did not give her permission to perform genetic analysis on her 9-year-old son.

Moreover, the genetic analysis performed has been focused only on PD-related genes, not considering the potential role of variants in other genes not related with parkinsonian phenotype. Therefore, this does not imply that p.G399S is responsible for the parkinsonian phenotype as a single mutation or in a complex genetic network.

Anyway, this novel finding suggests that 22q deletion is not the unique PD-related genetic alterations in patients carrying 22q11.2 deletions and PD, opening new avenues on potential associations between 22qdel patients and other genetic variants.

## 5. Conclusions

In this report, a mechanism that links DGs with parkinsonian features is proposed. In fact, to improve our knowledge about the role of concomitant mutations in patients with 22q11.2 deletions and EOPD, molecular testing considering all known PD-related genes is mandatory, which should now be included on the first rank PARK13 analysis.

This could help to decipher whether the 22q11.2 deletion links various neuropsychiatric disorders including schizophrenia. At the same time, a wide PD genes screening is expected to predict whether a single molecular mechanism links 22q11.2 deletion to PARK13 to produce a final common pathway or whether genetic defects other than PARK13 may be involved, alone or in combination with PARK13, to DGs phenotypes when coexisiting with 22q11.2 deletions [23].

At this time, clinicians should be alerted about the chance to detect 22q11.2 deletions in patients with early-onset PD or possessing clinical features which are typically associated with the chromosome 22q11.2 deletion syndrome or both. Patients known to carry a 22q11.2 deletion should be monitored for the development of parkinsonian symptoms.

## Figures and Tables

**Table 1:** List of sequence variants identified.

Locus	Gene	Genotype	HGVSc	HGVSp	SIFT	PolyPhen	Class	Criteria	dbSNP ID
PARK8	LRRK2	hom	NM_198578.3:c.149G>A	NP_940980.3:p.Arg50His	Tolerated (0.61)	Benign (0)	1	BA1	rs2256408
		het	NM_198578.3:c.2857T>C	NM_198578.3:c.2857T>C (p.=)			1	BA1	rs7966550
		het	NM_198578.3:c.7155A>G	NM_198578.3:c.7155A>G (p.=)			1	BA1	rs33962975
		het	NM_198578.3:c.7190T>C	NP_940980.3:p.Met2397Thr	Tolerated (0.71)	Benign (0.001)	1	BA1	rs3761863

PAKK2	PRKN	het	NM_004562.2:c.1138G>C	NP_004553.2:p.Val380Leu	Tolerated (0.83)	Benign (0.001)	1	BA1	rs1801582

PARK17	VPS35	hom	NM_018206.4:c.1938C>T	NM_018206.4:c.1938C>T (p.=)			1	BA1	rs168745

PARK6	PINK1	het	NM_032409.2:c.189C>T	NM_032409.2:c.189C>T (p.=)			1	BA1	rs45530340
		hom	NM_032409.2:c.388-7A>G				1	BA1	rs2298298
		hom	NM_032409.2:c.960-5G>A				1	BA1	rs3131713

PARK9	ATP13A2	hom	NM_022089.2:c.3516G>A	NM_022089.2:c.3516G>A (p.=)			1	BA1	rs3170740
		hom	NM_022089.2:c.3192C>T	NM_022089.2:c.3192C>T (p.=)			1	BA1	rs9435659
		hom	NM_022089.2:c.2970G>A	NM_022089.2:c.2970G>A (p.=)			1	BA1	rs761421
		hom	NM_022089.2:c.2637C>T	NM_022089.2:c.2637C>T (p.=)			1	BA1	rs9435662
		hom	NM_022089.2:c.1815C>T	NM_022089.2:c.1815C>T (p.=)			1	BA1	rs2076603

PARK15	FBXO7	hom	NM_012179.3:c.122+272T>G				1	BA1	rs8137714
		hom	NM_012179.3:c.345G>A	NP_036311.3:p.Met115Ile	Tolerated (0.19)	Benign (0)	1	BA1	rs11107
		het	NM_012179.3:c.540A>G	NM_012179.3:c.540A>G (p.=)			1	BA1	rs41311141
		hom	NM_012179.3:c.949C>T	NM_012179.3:c.949C>T (p.=)			1	BA1	rs9726

SLC52A3	hom	NM_033409.3:c.1233T>C	NM_033409.3:c.1233T>C (p.=)				1	BA1	rs910857
		hom	NM_033409.3:c.765C>T	NM_033409.3:c.765C>T (p.=)			1	BA1	rs3746805
		het	NM_033409.3:c.321C>T	NM_033409.3:c.321C>T (p.=)			1	BA1	rs3746808

PARK10	ELOVL4	het	NM_022726.3:c.895A>G	NP_073563.1:p.Met299Val	Tolerated (0.92)	Benign (0)	1	BA1	rs3812153

PARK11	GIGYF2	hom	NM_001103147.1:c.3003A>G	NM_001103147.1:c.3003A>G (p.=)			1	BA1	rs3816334
		hom	NM_001103147.1:c.3524-9G>A				1	BA1	rs2305137
		hom	NM_001103147.1:c.3693_3695delACA	NP_001096617.1:p.Gln1232del			1	BA1	rs10555297

PARK13	HTRA2	het	NM_013247.4:c.1195G>A	NP_037379.1:p.Gly399Ser	Deleterious (0.04)	Probably damaging (0.924)	4	PS3, PP3, PP5	rs72470545

PARK18	EIF4G1	hom	NM_001194947.1:c.502A>G	NP_001181876.1:p.Thr168Ala	Tolerated (0.81)	Benign (0)	1	BA1	rs13319149
		hom	NM_001194947.1:c.1315A>G	NP_001181876.1:p.Met439Val	Tolerated (0.36)	Benign (0)	1	BA1	rs2178403
		hom	NM_001194947.1:c.3974+9A>C				1	BA1	rs939317

For each variant, the following are reported: locus, gene, genotype detected, HGVS nomenclature (coding and protein, resp., HGVSc and HGVSp), SIFT and PolyPhen prediction (if available), and SNP ID. Furthermore, variants has been classified according to ACMG guidelines in five classes according to Richards et al. [24]. Class and criteria are listed in dedicated columns (BA1: stand-alone evidence of benign impact; PS3: criteria number 3 of strong evidence of pathogenicity; PP3 and PP5: criteria numbers 3 and 5, respectively, of supporting evidence of pathogenicity) [24].

## Data Availability

All data reported in this manuscript are included within the article. Raw data, if required, are available on request.
