# Erratum to “Mitochondrial Serine Protease HTRA2 p.G399S in a Female with Di George Syndrome and Parkinson's Disease”

**DOI:** 10.1155/2018/5956437

**Published:** 2018-09-09

**Authors:** Stefano Gambardella, Rosangela Ferese, Simona Scala, Stefania Carboni, Francesca Biagioni, Emiliano Giardina, Stefania Zampatti, Nicola Modugno, Francesco Fabbiano, Francesco Fornai, Diego Centonze, Stefano Ruggieri

**Affiliations:** ^1^IRCCS Neuromed, Pozzilli, Italy; ^2^Molecular Genetics Laboratory UILDM, Santa Lucia Foundation, Rome, Italy; ^3^Department of Biomedicine and Prevention, University of Rome “Tor Vergata”, Rome, Italy; ^4^Department of Translational Research and New Technologies in Medicine and Surgery, University of Pisa, Pisa, Italy

In the article titled “Mitochondrial Serine Protease HTRA2 p.G399S in a Female with Di George Syndrome and Parkinson's Disease” [1], the name of the sixth author was given incorrectly as Giardina Emiliano. The author's name should have been written as Emiliano Giardina. The revised authors' list is shown above.
